# Metal-Organic Framework Templated Synthesis of Ultrasmall Catalyst Loaded ZnO/ZnCo_2_O_4_ Hollow Spheres for Enhanced Gas Sensing Properties

**DOI:** 10.1038/srep45074

**Published:** 2017-03-22

**Authors:** Won-Tae Koo, Seon-Jin Choi, Ji-Soo Jang, Il-Doo Kim

**Affiliations:** 1 Department of Materials Science and Engineering and Korea Advanced Institute of Science and Technology, Daejeon 305-701, Republic of Korea; 2 Applied Science Research Institute, Korea Advanced Institute of Science and Technology, Daejeon 305-701, Republic of Korea

## Abstract

To achieve the rational design of nanostructures for superior gas sensors, the ultrasmall nanoparticles (NPs) loaded on ternary metal oxide (TMO) hollow spheres (HS) were synthesized by using the polystyrene (PS) sphere template and bimetallic metal-organic framework (BM-MOFs) mold. The zinc and cobalt based zeolite imidazole frameworks (BM-ZIFs) encapsulating ultrasmall Pd NPs (2–3 nm) were assembled on PS spheres at room temperature. After calcination at 450 °C, these nanoscale Pd particles were effectively infiltrated on the surface of ZnO/ZnCo_2_O_4_ HSs. In addition, the heterojunctions of Pd-ZnO, Pd-ZnCo_2_O_4_, and ZnO-ZnCo_2_O_4_ were formed on each phase. The synthesized Pd-ZnO/ZnCo_2_O_4_ HSs exhibited extremely high selectivity toward acetone gas with notable sensitivity (S = 69% to 5 ppm at 250 °C). The results demonstrate that MOF driven ultrasmall catalyst loaded TMO HSs were highly effective platform for high performance chemical gas sensors.

Metal oxides, such as SnO_2_, WO_3_, ZnO, TiO_2_ and Co_3_O_4_ based chemiresistive gas sensors have attracted great attention for applications in the detection of volatile organic compounds due to their low cost, real-time detection, and portability^1^,^2^. However, the low selectivity is still a big obstacles for metal oxide based gas sensors, because they are operated by the surface reaction of the target gas with the sensing materials. According to the reported literature so far, the highly effective ways to improve the performance of metal oxide based gas sensors are to increase the surface area and functionalizing the catalyst^3^,^4^. To increase the reaction sites, hollow nanostructures have attracted much attention due to their high surface area and gas permeability^5^,^6^,^7^. In addition, the noble metals such as Pd, Pt, and Au have been commonly used for the catalytic sensitization of chemical gas sensors^8^,^9^. However, it is still challenging to detect an analyte gas with high accuracy in highly humid atmosphere. To utilize the early diagnosis of disease, the sensor should detect the biomarker gas with high humidity in exhaled breath under sub-ppm level^10^. For example, the concentration of acetone which is a known biomarker gas for diabetes, is increased to 1.8 parts per million (ppm) in breath of diabetes patients, comparing with that (0.3–0.9 ppm) of healthy human^11^. To achieve the highly accurate detection of biomarker gas in exhaled breath with outstanding selectivity and superior sensitivity, the new sensing materials having high surface area and ultrasmall catalyst should be investigated.

Recently, metal-organic frameworks (MOFs), synthesized by assembly of metal ions and organic ligands, have received great attention due to their fascinating properties such as ultra-high surface area, incredibly high porosity, and diverse structures^12^,^13^,^14^. To facilitate these advantages of MOFs, a number of researchers have attempted to explore various applications, including catalysis^15^, drug delivery^16^, energy device^17^, and gas adsorption and storage^18^,^19^,^20^. Among various structure of MOFs, MOF based hollow spheres (HSs) have attracted broad interest because of their distinctive features such as high surface area, high gas permeability, low density, and high loading capacity^21^. Lee *et al*. synthesized hollow zeolite imidazole framework (ZIF) microspheres using polystyrene (PS) microsphere templates which were finally removed by organic solvent such as dimethylformamide^22^. Zhang *et al*. reported hollow ZIF nanospheres using PS sphere template as a highly efficient catalyst for cycloaddition reactions^23^. Besides, a number of researchers have been interested in MOF based HS as a catalyst^24^,^25^,^26^. However, the study of MOF based HS is still in infancy due to the limiting factors such as poor chemical stability, a micrometer size range, a wide size distribution, a simple shell architecture, and uncertain shapes.

One of the remarkable advantages of the MOF is its unique infiltration property of nanoscale metal particles. In other words, noble metal nanoparticles (NPs) can be encapsulated in the cavity of the MOF^27^,^28^. The growth of noble metal NPs was suppressed to less than the pore size by embedding them in the MOF, thereby limiting the particle size under 10 nm. These ultrasmall NPs are well-distributed in the cavities of MOFs, and they can create NP network in MOF. Therefore, a number of researchers have attempted to utilize the ultrasmall NPs in the MOF. Hermes *et al*. reported metal loaded MOF-5 as a catalyst for cyclooctene hydrogenation and CO oxidation by infiltrating of metal atoms using metal organic chemical vapor deposition^27^. Lu *et al*. suggested the NP encapsulation in the cavity of ZIF-8 using polymer surfactants to control the size, shape and composition of NPs, and they investigated the catalytic activity of Pt or Au loaded ZIF-8 as a hydrogenation catalyst^28^. Although some papers have been reported for various applications of metal loaded MOF, the application of the NP encapsulated MOF has not been explored in metal oxide based chemical gas sensors.

Herein, we suggest MOF derived ultrasmall catalyst loaded ternary metal oxide (TMO) HS structure from bi-metallic ZIF (BM-ZIF) with the PS sacrificial templates. As a template, the MOF based HSs containing ultrasmall NPs are calcined in air to synthesize metal oxide based sensing materials. As previously reported, the BM-ZIF can be oxidized to TMO by the calcination^29^. In addition, the organic ligands of MOFs and PS spheres are decomposed during the calcination, which lead to the creation of meso- and macro-pores in the surface of TMO HSs. Furthermore, ultrasmall NPs are well-distributed in the TMO HSs. Therefore, the MOF templated ultrasmall catalyst loaded TMO HSs have powerful advantages such as large reaction sites, high gas permeability, and high catalytic activities, which are crucial properties for the development of superior gas sensors. To utilize these benefits of the novel structure and expand the application of MOF, we propose the ultrasmall Pd catalyst loaded ZnO/ZnCo_2_O_4_ HSs (Pd-ZnO/ZnCo_2_O_4_ HSs) from Zn and Co based BM-ZIF template. The hollow structure synthesized by PS template can provide easy diffusion of analytic gases and the surface reaction in the inside/outside of sensing materials, resulting in the improvement of the chemical gas sensing performances. In addition, the ternary spinel ZnCo_2_O_4_ has more hole concentrations than spinel Co_3_O_4_, which leads to the increase in resistance change^30^. Moreover, the creation of the heterojunction by intentionally separating ZnO phase in the final product can improve the sensing performance by additional resistance changes in sensing materials. Furthermore, the well-dispersed and ultrasmall Pd NPs in TMO HSs can fully utilize the catalytic reactions and significantly enhance the sensing characteristics. To the best of our knowledge, this unique structure derived by MOFs has not yet been reported.

## Results and Discussion

### Synthesis of Pd-ZnO/ZnCo_2_O_4_ HSs

**Figure 1:**
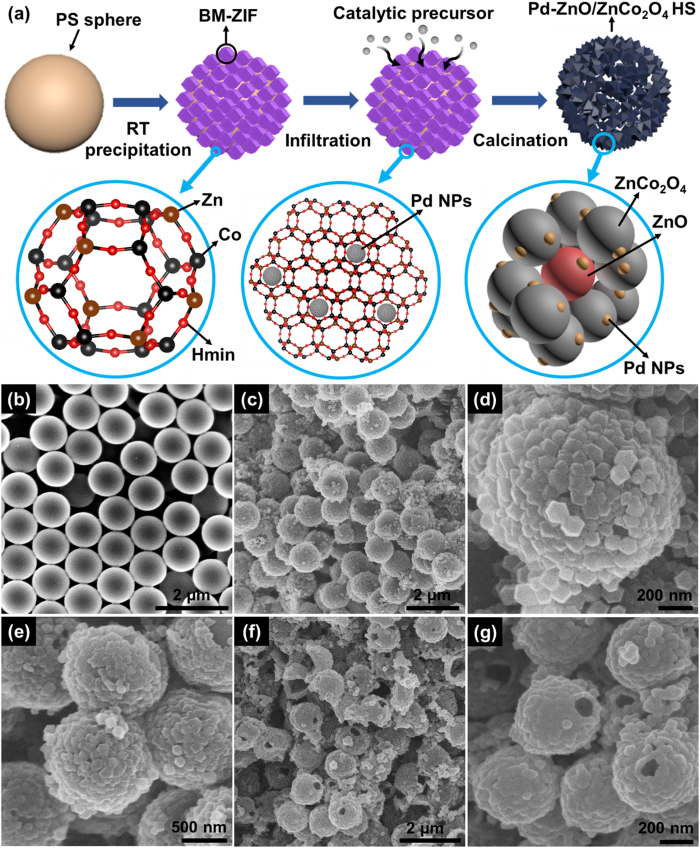
(**a**) Schematic illustration of synthetic process for the Pd-ZnO/ZnCo_2_O_4_ HSs. SEM images of (**b**) PS sphere template, (**c**) BM-ZIF/PS, (**d**) magnified image of BM-ZIF/PS (**e**) Pd-BM-ZIF/PS, (**f**) Pd-ZnO/ZnCo_2_O_4_ HSs, and (**g**) magnified image of Pd-ZnO/ZnCo_2_O_4_ HSs.

Figure 1a illustrates the synthetic process of Pd-ZnO/ZnCo_2_O_4_ HSs by using PS and MOF templates. Firstly, PS spheres were dispersed in methanol (MeOH). Then, Co and Zn precursors were added in the solution. The positively charged Co and Zn ions were easily coated on the surface of PS spheres due to the negative charge on the PS sphere^31^. After the solution was mixed for 30 min, the 2-methylimidazole (Hmin), which is an organic ligand, dissolved in MeOH solution was added to the mixture solution. Through the precipitation at the room temperature (RT), the BM-ZIFs were grown on the surface of the PS spheres^32^. After purification of BM-ZIFs on PS sphere (BM-ZIF/PS), Pd catalysts were encapsulated in the cavity of BM-ZIF by infiltration method in solution, and reduced by NaBH_4_ reduction^33^. Finally, these Pd-encapsulated BM-ZIF/PSs (Pd-BM-ZIF/PSs) were calcined at 450 °C for 1 h, which generates the Pd-ZnO/ZnCo_2_O_4_ HSs.

The scanning electron microscopy (SEM) analysis was performed to investigate the morphology and structure of Pd-ZnO/ZnCo_2_O_4_ HSs. The diameter of PS sphere used in the experiment was 1 μm (Fig. 1b). After RT precipitation process, BM-ZIFs were uniformly coated on the PS sphere, and the size of BM-ZIF was confirmed to be about 100 nm (Fig. 1c and d). To convince the formation of BM-ZIF/PS, powder X-ray diffraction (PXRD) and N_2_ adsorption/desorption analysis were also investigated (Fig. S1). The PXRD patterns of BM-ZIF/PSs are well-matched with the simulated peaks of BM-ZIFs. In addition, the N_2_ adsorption analysis exhibited micro-porous structure with high surface area (1147 m^2^/g). After Pd encapsulation, there were no significant changes in the SEM images (Fig. 1e). Because the size of the settled Pd NPs in the BM-ZIF was determined as ultrasmall (2–3 nm)^33^, it could not be analyzed using SEM. The Pd-BM-ZIFs enclosed the surface of PS spheres act as templates, so that it is possible to synthesize HS structure after high-temperature heat-treatment. Moreover, the BM-ZIFs are oxidized to ZnO and ZnCo_2_O_4_ during the calcination at 450 °C. As a result, the ZnO/ZnCo_2_O_4_ HSs were synthesized, and ultrasmall Pd NPs were functionalized on the ZnO/ZnCo_2_O_4_ matrix. The organic compounds of BM-ZIFs and PS spheres were decomposed during the calcination process. Therefore, the size of Pd-ZnO/ZnCo_2_O_4_ HSs was reduced to 400 nm (Fig. 1f). In addition, the meso-pores were created on the Pd-ZnO/ZnCo_2_O_4_ HSs during the decomposition of the organic ligands in the BM-ZIF (Fig. S2). Moreover, the macro-pores were also generated on the surface of the Pd-ZnO/ZnCo_2_O_4_ HSs (Fig. 1g). These macro-pores were formed at a certain position where BM-MOF is not densely grown on the PS sphere. The meso- and macro-pores in the Pd-ZnO/ZnCo_2_O_4_ HSs not only increase surface area, but also facilitate gas diffusion inside of the product^4^. As a result, it is possible to provide a large reaction site in case of hollow spheres. However, the existence of Pd NPs was not confirmed in the SEM images of Pd-ZnO/ZnCo_2_O_4_ HSs.

**Figure 2:**
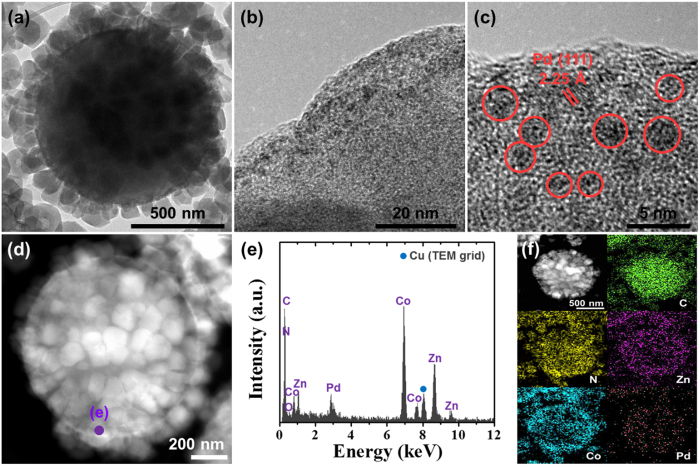
(**a**) TEM image of Pd-ZIF/PS, (**b**,**c**) HRTEM images of Pd-ZIF/PS, (**d**) STEM images of Pd-BM-ZIF/PS, (**e**) EDS spectrum of Pd-ZIF/PS, and (**f**) EDS elemental mapping images of Pd-ZIF/PS.

In order to confirm the presence of the ultrasmall Pd NPs in the BM-ZIF/PSs, we conducted transmission electron microscopy (TEM) analysis using Pd-BM-ZIF/PSs. The TEM image of Pd-BM-ZIF/PS revealed that Pd-BM-ZIFs were densely surrounded on the PS sphere (Fig. 2a). In high resolution TEM (HRTEM) of Pd-BM-ZIF/PS, the small black spheres presumed to Pd NPs were presented in the surface of BM-ZIF (Fig. 2b). Pd^2+^ ions were diffused into the cavities of BM-ZIF in the solution, where most of Pd NPs were settled on the surface of BM-ZIF. In further magnified image, the Pd NPs were confirmed with the crystal plane of Pd (111) (Fig. 2c). Surprisingly, the size of Pd NPs in the BM-ZIF/PS was identified as about 2–3 nm. To concretely verify the existence of Pd NPs, energy dispersive X-ray spectroscopy (EDS) analysis was carried out by TEM at purple sphere point in Fig. 2d. Pd and other basic elements of BM-ZIF/PS were detected by EDS point analysis (Fig. 2e). In addition, EDS elemental mapping images show that the Pd-BM-ZIF, consisted of C, N, Zn, and Co, was well-grown on the PS sphere and the Pd NPs were well-dispersed in the Pd-BM-ZIF/PS (Fig. 2f).

Thermal gravimetric analysis (TGA) of Pd-ZnO/ZnCo_2_O_4_ HSs was conducted to investigate the thermal behavior of Pd-BM-ZIF/PS and the thermal decomposition of organic molecules (Fig. S3). At first, there was a noticeable weight loss at 280 °C. This weight degradation is related to the decomposition of PS spheres because PS spheres are normally decomposed in the temperature range of 200–450 °C^34^. At 350 °C, there was the additional decrease of weight due to the burning-out of organic molecules in BM-ZIF, consistent with a previous study of thermal behavior of BM-ZIF^19^. The TGA results reveal that the calcination process at 450 °C for 1 h in air is enough to eliminate the organic compounds and PS spheres in Pd-BM-ZIF/PSs.

**Figure 3:**
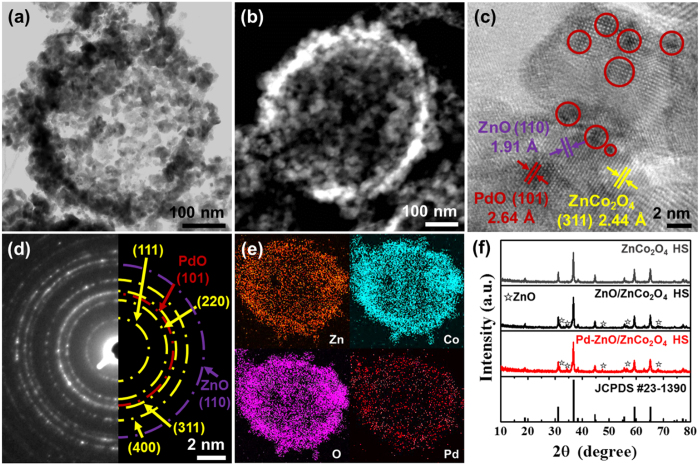
(**a**) TEM image of Pd-ZnO/ZnCo_2_O_4_ HS, (**b**) STEM image of Pd-ZnO/ZnCo_2_O_4_ HS, (**c**) HRTEM image of Pd-ZnO/ZnCo_2_O_4_ HS, (**d**) SAED pattern of Pd-ZnO/ZnCo_2_O_4_ HS, (**e**) EDS elemental mapping analysis of Pd-ZnO/ZnCo_2_O_4_ HS, and (**f**) PXRD analysis of ZnCo_2_O_4_ HSs, ZnO/ZnCo_2_O_4_ HSs, and Pd-ZnO/ZnCo_2_O_4_ HSs.

After calcination at 450 °C for 1 h, the TEM analysis was also investigated to clearly observe the structure of the Pd-ZnO/ZnCo_2_O_4_ HSs. The Pd-ZnO/ZnCo_2_O_4_ synthesized by the PS sphere and BM-ZIF templates exhibited highly porous hollow sphere structures (Fig. 3a). As discussed in Fig. 2, there were a number of meso- and macro-pores in Pd-ZnO/ZnCo_2_O_4_ HSs. In the dark field scanning TEM (STEM) images, numerous pores were noticeably observed (Fig. 3b). In addition, the thickness of the wall in Pd-ZnO/ZnCo_2_O_4_ HSs was confirmed as about 35 nm. Moreover, the HRTEM image reveals the (311), (110), and (101) crystal plane of ZnCo_2_O_4_ (spinel), ZnO (hexagonal), and PdO (tetragonal), respectively, which correspond to lattice distance of 2.44 Å, 1.91 Å, and 2.64 Å (Fig. 3c). Furthermore, the size of PdO NPs was determined to be 2–3 nm. Selective area electron diffraction (SAED) patterns show the polycrystalline ZnCo_2_O_4_ HSs with the (111), (220), (311), and (400) crystal plane (Fig. 3d). In addition, the crystal plane of ZnO (110) and PdO (101) were displayed in the SAED pattern. The SAED patterns of PdO were weak due to the low concentration of PdO in the Pd-ZnO/ZnCo_2_O_4_ HSs. To further identify the presence of the Pd NPs in the Pd-ZnO/ZnCo_2_O_4_ HSs, we performed EDS elemental mapping analysis by TEM (Fig. 3e). The EDS mapping images demonstrate that Pd were properly distributed in the Pd-ZnO/ZnCo_2_O_4_ HSs. These ultrasmall and well-dispersed Pd NPs can promote the surface reaction on ZnO/ZnCo_2_O_4_ HSs.

### Microstructure and chemical analysis of Pd-ZnO/ZnCo_2_O_4_ HSs

The crystal structure of Pd-ZnO/ZnCo_2_O_4_ HSs was investigated by PXRD analysis (Fig. 3f). We synthesized the ZnCo_2_O_4_ HSs as a control sample to demonstrate the ZnO phase separation. According to the previous article, the phase of ZnCo_2_O_4_ cubes synthesized by BM-ZIF templates changes with respect to the molar ratio of Zn and Co in the BM-ZIF^29^. In case of the molar ratio of Zn and Co for 1:3, ZnCo_2_O_4_ cubes exhibited pure ZnCo_2_O_4_ spinel structure. If the molar ratio of Zn to Co was 1:2, the hexagonal ZnO phase was separated because the Zn atoms were not incorporated into the Co_3_O_4_ lattice. Therefore, in the same way, we can synthesize pure ZnCo_2_O_4_ HSs and ZnO/ZnCo_2_O_4_ HSs by controlling the molar ratio of Zn and Co in BM-ZIF. As a result, the BM-ZIF/PS which Zn/Co molar ratio was 0.33 are converted to pure ZnCo_2_O_4_ spinel structure (JCPDS no. 23–1390). On the contrary, when the molar ratio of Zn/Co was changed to 0.5, the calcined products show the major spinel phase of ZnCo_2_O_4_ with the secondary phase of hexagonal ZnO (JCPDS no. 65–3411). Similarly, the XRD peaks of Pd-ZnO/ZnCo_2_O_4_ HSs indicated strong intensity of spinel ZnCo_2_O_4_ phase and weak intensity of hexagonal ZnO phase. Unfortunately, the Pd related peaks were not observed in the XRD data because the loading amounts of Pd in Pd-ZnO/ZnCo_2_O_4_ HSs were beyond the limit of detection of XRD analysis. The XRD analysis confirms that the phase separation of ZnO has occurred on the ZnCo_2_O_4_ matrix, and forms heterojunction in Pd-ZnO/ZnCo_2_O_4_ HSs.

**Figure 4:**
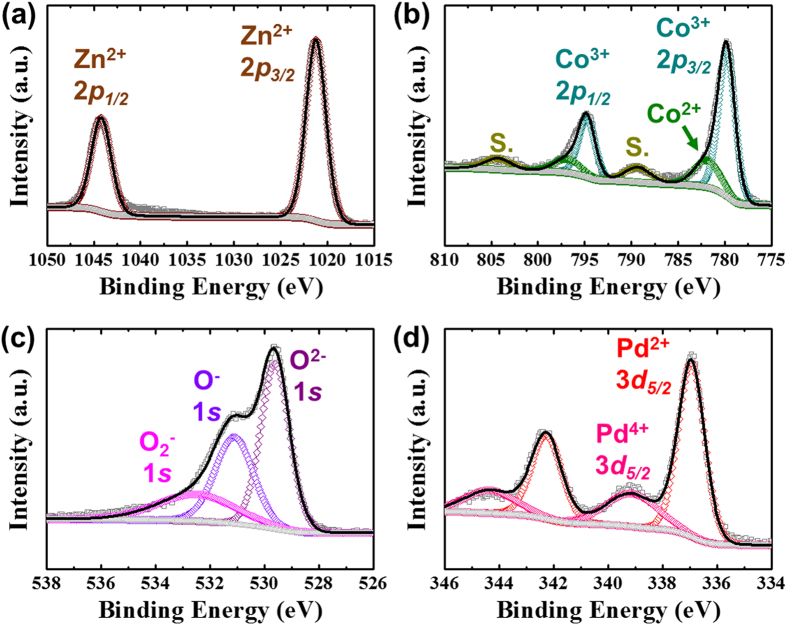
XPS analysis using high resolution spectrum of Pd-ZnO/ZnCo_2_O_4_ HSs in the vicinity of (**a**) Zn 2*p*, (**b**) Co 2*p*, (**c**) O 1 *s*, and (**d**) Pd 3*d*.

In addition, the chemical bonding states of Pd-ZnO/ZnCo_2_O_4_ HSs were verified by using X-ray photoelectron spectroscopy (XPS) (Fig. 4). The high resolution spectrum of Zn 2*p* in Pd-ZnO/ZnCo_2_O_4_ HSs exhibited two characteristic peaks of 2*p*_1/2_ and 2*p*_*3/2*_ at binding energies of 1021.2 eV and 1044.2 eV, corresponding to the binding energy of Zn^2+^ state (Fig. 4a)^35^. The Zn^2+^ state revealed that the Zn atoms in Pd-BM-ZIF/PS were oxidized to ZnO and ZnCo_2_O_4_. The Co 2*p* spectrum presented the dominant peaks at 779.4 eV for Co^3+^ 2*p*_*3/2*_, and at 794.8 eV for Co^3+^ 2*p*_*1/2*_ (Fig. 4b)^35^. In addition, the minor peaks of Co^2+^ 2*p*_*3/2*_, Co^2+^ 2*p*_*1/2*_, and satellite peaks were also observed in the high resolution spectra of Co 2*p*. These results are well-matched with other literatures on XPS analysis of spinel ZnCo_2_O_4_^35^,^36^,^37^. Therefore, we clearly confirmed the formation of spinel ZnCo_2_O_4_ during the calcination. Further, oxygen peaks in O 1 *s* spectrum showed three oxygen states, which were located at 529.6 eV for O^2−^ 1 *s*, 531.1 eV for O^−^ 1 *s*, and 532.5 eV for O_2_^−^ 1 *s*, as shown in Fig. 4c. The O^2−^ state is related to oxygen in ZnO and ZnCo_2_O_4_, and the O^−^ and O_2_^−^ state are originated from the chemisorbed oxygen species on the surface of Pd-ZnO/ZnCo_2_O_4_ HSs^35^. Pd atoms loaded on BM-ZIF/PS were also oxidized to PdO and PdO_2_ during the heat-treatment steps. Therefore, the chemical states of Pd were Pd^2+^ and Pd^4+^, corresponding to PdO and PdO_2_ respectively (Fig. 4d)^38^.

### Gas sensing characteristics

**Figure 5:**
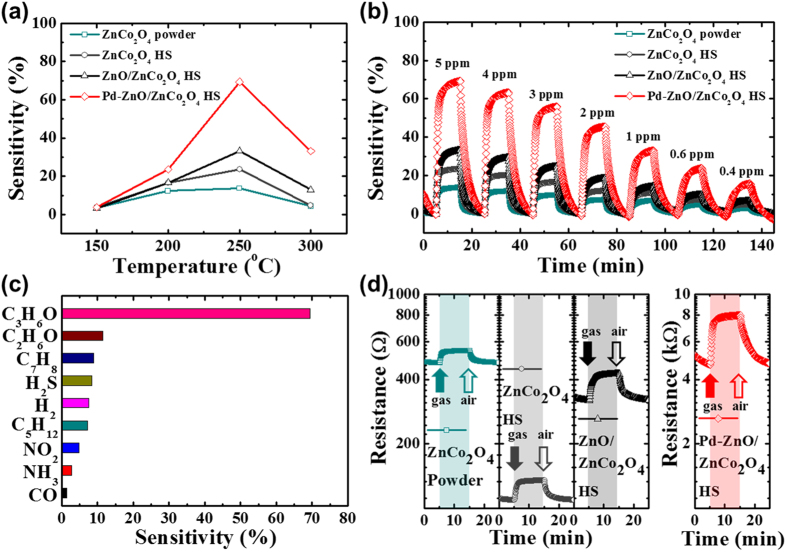
(**a**) Temperature dependent acetone sensing characteristics to 5 ppm in a temperature range of 150–300 °C, and (**b**) dynamic acetone sensing transition in the concentration range of 0.4–5 ppm at 250 °C of ZnCo_2_O_4_ powders, ZnCo_2_O_4_ HSs, ZnO/ZnCo_2_O_4_ HSs, and Pd-ZnO/ZnCo_2_O_4_ HSs. (**c**) selective acetone detection characteristics of Pd-ZnO/ZnCo_2_O_4_ HSs, (**d**) dynamic resistance transition properties of ZnCo_2_O_4_ powders, ZnCo_2_O_4_ HSs, ZnO/ZnCo_2_O_4_ HSs, and Pd-ZnO/ZnCo_2_O_4_ HSs toward 5 ppm of acetone at 250 °C.

To evaluate the highly selective sensing properties of Pd-ZnO/ZnCo_2_O_4_ HSs, we prepared chemiresistive gas sensors using ZnCo_2_O_4_ powders, ZnCo_2_O_4_ HSs, ZnO/ZnCo_2_O_4_ HSs, and Pd-ZnO/ZnCo_2_O_4_ HSs. ZnCo_2_O_4_ powders were synthesized by the direct calcination of BM-ZIF as a control sample (Fig. S4). In addition, ZnCo_2_O_4_ HSs and ZnO/ZnCo_2_O_4_ HSs were prepared to demonstrate the effect of heterojunction and ultrasmall Pd NPs. The acetone gas (C_3_H_6_O), a biomarker in exhaled breath for diabetes^39^, sensing characteristics were investigated at a highly humid atmosphere (90% RH) to verify the potential application of early diagnosis of diabetes using exhaled breath analysis, which is a harsh sensing environment because the water vapor can decrease the sensing properties^40^. First of all, the sensitivity of acetone sensing was optimized toward the operating temperature (150–300 °C) and the concentration of catalyst (0.5–3.0 mg). Herein, sensitivity or response (S) was defined as (*R*_*gas*_/*R*_*air*_ − 1) × 100. As a result, the acetone sensing response exhibited highest sensitivity at 250 °C (Fig. 5a). In addition, the Pd-ZnO/ZnCo_2_O_4_ HSs synthesized by using 2.0 mg of Pd precursors during a catalyst encapsulation step exhibited highest response in comparison with other samples (Fig. S5a). Then, the humidity effect of the sensors was investigated. The baseline resistance of Pd-ZnO/ZnCo_2_O_4_ HSs was increased at a higher humidity (Fig. S5b), because the adsorption of water vapor can donate the electrons to the sensing layers^41^ and these electrons are recombined with hole in the ZnCo_2_O_4_. Therefore, the hole accumulation layers decreased, leading to the decrease in the sensing properties (Fig. S5c). The dynamic acetone sensing characteristics were investigated for the concentration range of 0.4 ppm to 5 ppm at 250 °C (Fig. 5b). The Pd-ZnO/ZnCo_2_O_4_ HSs exhibited highest sensitivity (S = 69%) toward 5 ppm of acetone, while ZnCo_2_O_4_ powder (S = 14%), ZnCo_2_O_4_ HSs (S = 23%), and ZnO/ZnCo_2_O_4_ HSs (S = 33%) showed low sensitivity. In addition, Pd-ZnO/ZnCo_2_O_4_ HSs were detected at 400 ppb of acetone with noticeable sensitivity (S = 16%). The results showed that the functionalization of Pd NPs and ZnO on ZnCo_2_O_4_ HSs dramatically improved the response of acetone sensing. To investigate the selectivity of the Pd-ZnO/ZnCo_2_O_4_ HSs, we conducted additional sensing measurement toward various interfering gases such as ethanol (C_2_H_6_O), toluene (C_7_H_8_), hydrogen sulfide (H_2_S), hydrogen (H_2_), pentane (C_5_H_12_), nitrogen dioxide (NO_2_), ammonia (NH_3_), and carbon monoxide (CO) at 5 ppm level (Fig. 5c). The experimental results confirm that the Pd-ZnO/ZnCo_2_O_4_ HSs exhibited ultrahigh selectivity toward acetone (S = 69%) compared with other gases (S < 9%). Furthermore, the sensor showed stable response even during repeated measurements using Pd-ZnO/ZnCo_2_O_4_ HSs toward 5 ppm of acetone at 250 °C (Fig. S5d). The long term stability of Pd-ZnO/ZnCo_2_O_4_ HSs was also investigated by comparing the 6-month old samples with the 6-day old samples. Although the sensitivity of the sensors was decreased to 57% (Fig. S5e), the sensors showed stable sensitivity toward 5 ppm of acetone molecules during 20 cyclic measurements (Fig. S5f).

### Gas sensing mechanism

The acetone sensing mechanism of chemiresistive gas sensors was discussed in the previous articles^42^,^43^,^44^. When ZnCo_2_O_4_ HSs are exposed to air, the oxygen (O_2_) molecules are chemisorbed on the surface of ZnCo_2_O_4_ HSs and transform to O^2−^, O^−^, and O_2_^−^. As the ZnCo_2_O_4_ is a p-type semiconductor, the chemisorption of oxygen molecules deprived the electron in ZnCo_2_O_4_ and created the hole accumulation layer in the surface of the ZnCo_2_O_4_ HSs. So, when ZnCo_2_O_4_ HSs are exposed to reducing gas such as acetone, the reducing gas reacts with chemisorbed oxygen species (O^2−^, O^−^, and O_2_^−^) on the surface of the ZnCo_2_O_4_ HSs. This surface reaction causes the transfer of electron toward sensing materials, so that the hole accumulation layer of ZnCo_2_O_4_ HSs decreases. As a result, the resistance of sensing materials increases as shown in Fig. 5d.

**Figure 6:**
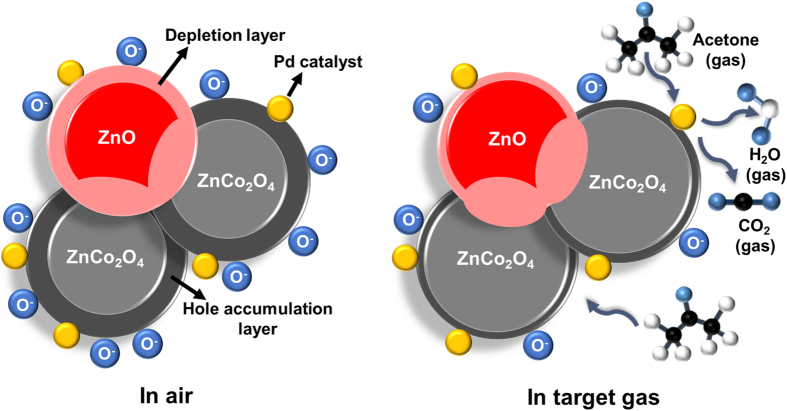
Schematic illustration of acetone sensing mechanism for Pd-ZnO/ZnCo_2_O_4_ HSs.

The high sensitivity of Pd-ZnO/ZnCo_2_O_4_ HSs toward acetone gas is ascribed by several factors. Firstly, hollow sphere structure is advantageous in comparison with powder structure from the gas diffusion aspects^3^. In case of ZnCo_2_O_4_ powder, the volatile organic compounds are difficult to diffuse into the sensing materials. On the contrary, in case of ZnCo_2_O_4_ HS, the analytic gases are easily diffused inside and outside of the sensing materials. Therefore, ZnCo_2_O_4_ HSs exhibited higher acetone sensing response (S = 14% to 5 ppm at 250 °C) than ZnCo_2_O_4_ powders (S = 23% to 5 ppm at 250 °C) because the reaction site increased (Fig. 5b). Secondly, the formation of p-n junction in the ZnCo_2_O_4_ HS enhanced sensing properties^45^. The separated n-type ZnO induced p-n junction in p-type ZnCo_2_O_4_, and the recombination between the electron in ZnO and the hole in ZnCo_2_O_4_ occurred. The recombination caused the decrease of hole concentration and the formation of depletion layer (Fig. 6), which increased the resistance of ZnO/ZnCo_2_O_4_ (Fig. 5d). When acetone molecules react with chemisorbed oxygen species on the surface of ZnO/ZnCo_2_O_4_, the effects of surface reactions are displayed in a different manner^45^. When ZnO/ZnCo_2_O_4_ is exposed to acetone, the ZnCo_2_O_4_ gains hole and the ZnO obtains electron from the surface reaction. The obtained electrons in the ZnO can recombine with holes in the ZnCo_2_O_4_ at the interface of ZnO and ZnCo_2_O_4_. This additional recombination causes the reduction of hole concentration in ZnCo_2_O_4_. Therefore, the resistance increase of ZnO/ZnCo_2_O_4_ HSs during the surface reaction is higher than that of ZnCo_2_O_4_ HSs (Fig. 5d). Lastly, the catalytic sensitization of Pd NPs dramatically improved the sensitivity of Pd-ZnO/ZnCo_2_O_4_ HSs. Pd is a well-known catalyst in chemiresisitve gas sensors as an electronic sensitizer^3^,^46^. The Pd NPs loaded in ZnO/ZnCo_2_O_4_ HSs can be oxidized to PdO and PdO_2_ in air, as shown in XPS analysis. When PdO and PdO_2_ NPs are exposed to acetone, the PdO and PdO_2_ NPs are reduced to Pd by donating electron back to ZnO/ZnCo_2_O_4_ HSs (Fig. 6). In addition, the Pd catalyst can promote the surface reaction by lowering the activation energy, with the following reactions (CH_3_COCH_3_ + O^−^ CH_3_COC^+^H_2_ + OH^−^ + e^−^ or CH_3_COCH_3_ + 2O^−^ CH_3_O^−^ + C^+^H_3_ + CO_2_ + 2e^−^)^44^. These additional electrons are recombined with hole in Pd-ZnO/ZnCo_2_O_4_ HSs, and the hole accumulation layer was remarkably decreased. Therefore, the acetone sensing response is dramatically increased by Pd catalyst (Fig. 5d). From the above reasons, the Pd-ZnO/ZnCo_2_O_4_ HSs exhibited high sensitivity and selectivity toward acetone. These superior chemical gas sensing results confirm the future possibility of early diagnosis of diabetes.

## Conclusions

In this work, the ultrasmall Pd NPs were effectively functionalized on the TMO HSs by the templating route using PS sphere and BM-MOF. The proposed Pd-ZnO/ZnCo_2_O_4_ HSs have a high surface area and gas permeability which come from the hollow structure. In addition, heterojunctions are created in the Pd-ZnO/ZnCo_2_O_4_ HSs by intentionally separating the ZnO phase. Furthermore, the very small and well-dispersed NPs can act as an effective catalyst for the surface reaction of ZnO/ZnCo_2_O_4_ HSs. For the potential applications, the chemical sensing properties of Pd-ZnO/ZnCo_2_O_4_ HSs were evaluated, and the results confirmed the remarkable sensitivity (S = 69% to 5 ppm at 250 °C) and outstanding selectivity toward acetone. The Pd-ZnO/ZnCo_2_O_4_ HSs exhibited high sensitivity toward acetone even in low ppm (S = 16% to 0.4 ppm at 250 °C). Moreover, the Pd-ZnO/ZnCo_2_O_4_ HSs can be used for the detection of acetone gas, with high stability even after a number of trials. These results demonstrate that MOF templated ultrasmall catalyst loaded TMO HS proves the possibility of high performance chemical gas sensors. In addition, the nanostructure of the proposed design concept can be also applicable to other devices requiring a high surface area and catalytic activity, such as oxygen evolution/reduction reaction catalysts, hydrogen evolution reaction catalysts, and Li-air batteries.

## Methods

### Materials

Cobalt nitrate hexahydrate (Co(NO_3_)_2_·6H_2_O), methanol (MeOH, 99.9%), ethanol (EtOH, 99.5%), and potassium tetrachloropalladate(II) (K_2_PdCl_4_) were purchased from Sigma-Aldrich. Zinc nitrate hexahydrate (Zn(NO_3_)_2_·6H_2_O, 98%), and 2-methylimidazole (Hmin, 99.0%) were purchased from Aldrich. PS latex microspheres (1 μm) dispersed in deionized (DI) water were purchased from Alfa Aesar. All chemicals were used without further purification.

### Synthesis of ZnCo_2_O_4_ powder

The Co and Zn based BM-ZIFs were firstly synthesized by mixing metal ions and Hmin at RT. 60 mg of Zn precursors and 180 mg of Co precursors were dissolved in 20 mL of MeOH. 540 mg of Hmin was also dissolved in 20 mL of MeOH. The solution was rapidly merged and stirred for 5 h in RT. Then, the mixed solution was purified by centrifugation and washed with EtOH. The obtained BM-ZIFs were calcined at 450 °C for 1 h with the ramping rate of 10 °C min^−1^.

### Synthesis of ZnCo_2_O_4_ HSs and ZnO/ZnCo_2_O_4_ HSs

As a sacrificial template, 0.9 mL of DI water containing 2.5 wt% PS spheres (1 μm) was dispersed in 20 mL of MeOH. Then, 60 mg of Zn precursors and 180 mg of Co precursors were added in the PS dissolved solution. After 30 min later, 20 mL MeOH containing 540 mg of Hmin was also poured into the PS sphere solution. The mixed solution was stirred using magnetic bar and precipitated for 5 h. The BM-ZIF/PS were obtained by the purification of the mixed solution. Finally, ZnCo_2_O_4_ HSs were synthesized by the calcination of the BM-ZIF/PS at 450 °C for 1 h (10 °C min^−1^). To synthesize ZnO/ZnCo_2_O_4_ HSs, 90 mg of Zn precursors was dissolved in the MeOH during the synthesis process of BM-ZIF/PS. The other experimental conditions are the same.

### Synthesis of Pd-ZnO/ZnCo_2_O_4_

To synthesize the Pd-ZnO/ZnCo_2_O_4_, we added the infiltration process of Pd ions before the calcination. 0.9 mL of PS sphere dispersed DI-water (2.5 wt%) and 540 mg of Hmin were dissolved separately in 20 mL MeOH. Then, 90 mg of Zn precursors and 180 mg of Co precursors were dissolved in the PS sphere solution. After 30 min, the two solutions were mixed and stirred for 5 h. The BM-ZIF/PS were purified using centrifugation and washing. The purified BM-ZIF/PS were added to 0.5–3.0 mg of K_2_PdCl_4_ dissolved DI-water (40 mL). The Pd metal ions in the cavity of BM-ZIF were reduced by NaBH_4_ solution (1.5 mg mL^−1^). Then, the Pd-BM-ZIF/PS were purified using centrifugation and washing in the same manner. Finally, Pd-ZnO/ZnCo_2_O_4_ HSs were synthesized by high-temperature heat-treatment using same calcination conditions.

### Sensor fabrication and gas sensing measurement

To evaluate the sensing performance of the synthesized product, we dispersed ZnCo_2_O_4_ powders, ZnCo_2_O_4_ HSs, ZnO/ZnCo_2_O_4_ HSs, and Pd-ZnO/ZnCo_2_O_4_ HSs in EtOH, respectively. These dispersed solutions were drop-coated on the sensor substrate (Al_2_O_3_, area: 2.5 mm × 2.5 mm, thickness 0.2 mm), respectively. Two parallel Au electrodes (width: 25 μm, gap size: 70 μm) were patterned on the front side and a Pt micro-heater on the back side of the sensor substrate. Gas sensing performances were investigated toward various analytic gases in the temperature range of 150–300 °C. To stabilize the sensor, the air was injected for 10 min in the chamber. Then, the sensor was exposed to analytic gas for 10 min. The concentration of gas was controlled to 400 ppb to 5 ppm, and the humidity was maintained at 90% RH. The selectivity toward target gas was investigated by the calculating the resistance changes of the sensors using acquisition system (34972, Agilent). The operating temperature was modulated by controlling the voltage of the Pt micro-heater patterned on the backside of the sensors using DC power supply (E3647A, Agilent).

### Characterization

The morphologies of samples were analyzed by field emission scanning electron microscopy (Nova230, FEI). The microstructure was investigated by field emission transmission electron microscopy (Tecnai G2 F30 S-Twin, FEI). The crystal structure was examined by powder X-ray diffraction (D/MAX-2500, Rigaku) analysis using Cu Kα radiation (λ = 1.5418 Å). The X-ray photoelectron spectroscopy (Sigma Probe, Thermo VG Scientific) analysis was conducted to investigate the chemical binding states. To confirm the pore distribution and the Brunauer–Emmett–Teller (BET) surface area, N_2_ adsorption/desorption isotherms (Tristar 3020, Micromeritics) were conducted at 77 K. Thermal gravimetric analysis (Labsys Evo, Setaram) was carried out to analyze the thermal behavior.

## Additional Information

**How to cite this article:** Koo, W.-T. *et al*. Metal-Organic Framework Templated Synthesis of Ultrasmall Catalyst Loaded ZnO/ZnCo_2_O_4_ Hollow Spheres for Enhanced Gas Sensing Properties. *Sci. Rep.* **7**, 45074; doi: 10.1038/srep45074 (2017).

**Publisher's note:** Springer Nature remains neutral with regard to jurisdictional claims in published maps and institutional affiliations.

## Supplementary Material

Supplementary Information
