# Supplementary material for: Metal-Organic Framework Templated Synthesis of Ultrasmall Catalyst Loaded ZnO/ZnCo2O4 Hollow Spheres for Enhanced Gas Sensing Properties
Source: Sci Rep. 2017 Mar 22;7:45074. doi: 10.1038/srep45074 (PMC5361158; doi:10.1038/srep45074)
Supplement: Supplementary Information [file srep45074-s1.pdf]

# Supporting Information

# Metal-Organic Framework Templated Synthesis of Ultrasmall Catalyst Loaded $ZnO/ZnCo_2O_4$ Hollow Spheres for Enhanced Gas Sensing Properties

Won-Tae Koo,[1] Seon-Jin Choi, [1,2] Ji-Soo Jang[1] and Il-Doo Kim*[1]

[1]Department of Materials Science and Engineering and [2]Applied Science Research Institute, Korea Advanced Institute of Science and Technology, Daejeon 305-701, Republic of Korea

[*] Corresponding author. Tel.: +82 42 350 3329; Fax: +82 42 350 3310.

E-mail address: idkim@kaist.ac.kr

**Table of Contents**

1. Characterization of BM-ZIF/PS

2. $N_2$ adsorption/desorption and pore size distribution of Pd-$ZnO/ZnCo_2O_4$ HSs

3. TG/DTG analysis of Pd-BM-ZIF/PS

4. SEM images of BM-ZIFs and $ZnCo_2O_4$ powders

5. Supplementary sensing characteristics

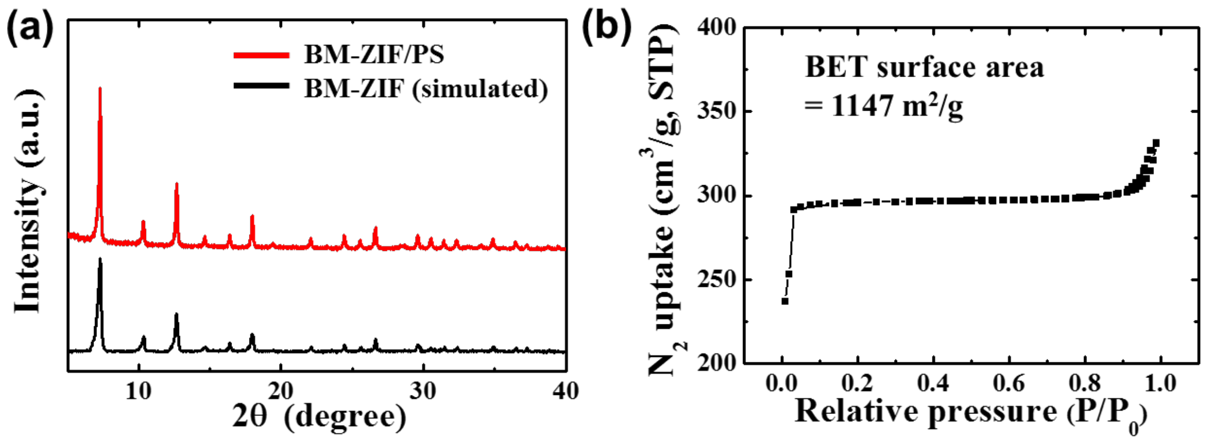


**Figure S1.** (a) PXRD analysis of BM-ZIF/PS, (b) $N_2$ adsorption/desorption analysis of BM-ZIF/PS.

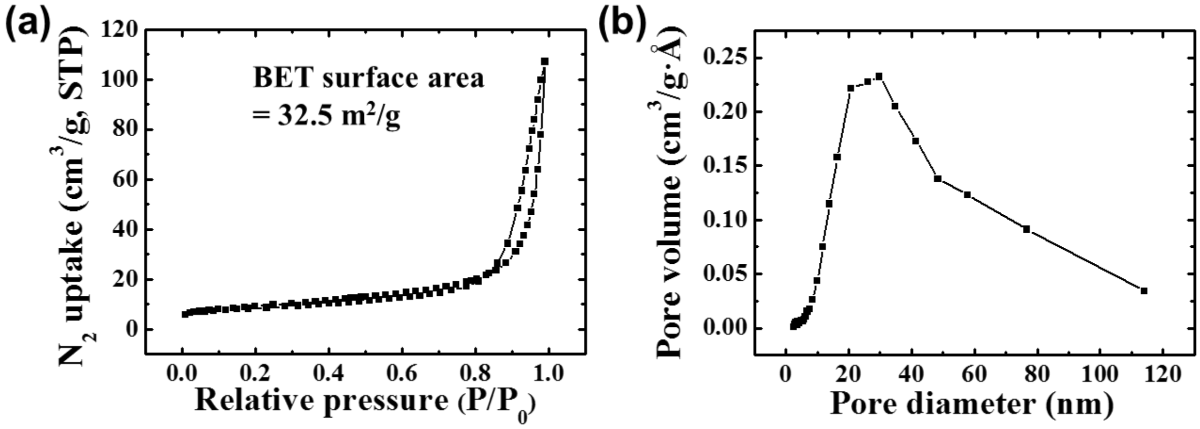


**Figure S2.** (a) $N_2$ adsorption/desorption isotherms of Pd-ZnO/$ZnCo_2O_4$ HSs, (b) pore size distribution of Pd-ZnO/$ZnCo_2O_4$ HSs.

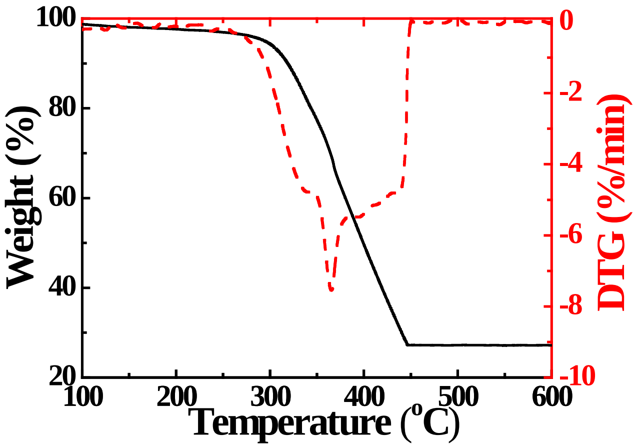


**Figure S3.** TGA and DTG analysis of the Pd-BM-ZIF/PS HSs in the temperature range of 100–600 °C under air atmosphere.

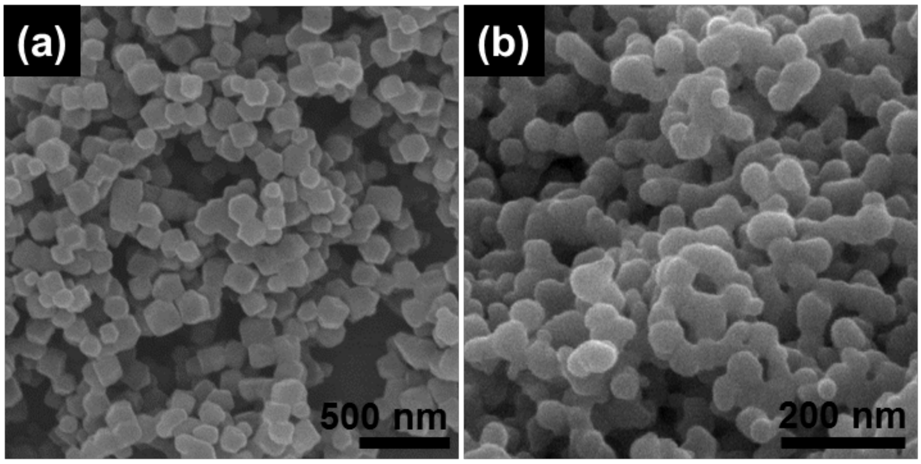


**Figure S4.** SEM images of (a) BM-ZIF, and (b) $ZnCo_2O_4$ powders calcined at 450 °C.

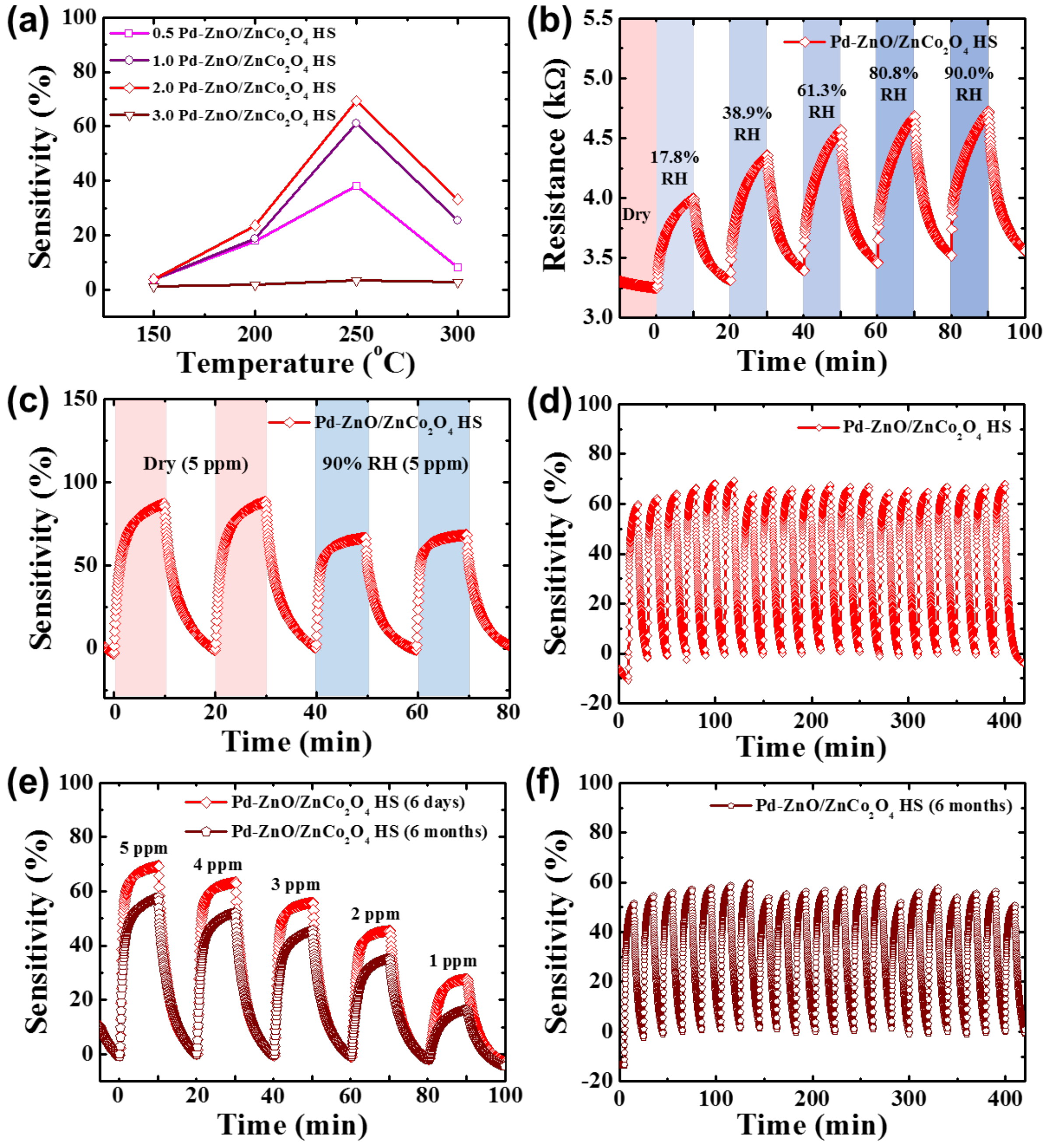


**Figure S5.** (a) Acetone sensing characteristics to 5 ppm in different concentration of Pd NPs in Pd-ZnO/$ZnCo_2O_4$ HSs at the temperature range of 150–300 °C, (b) Dynamic resistance of Pd-ZnO/$ZnCo_2O_4$ HSs in the different humidity conditions at 250 °C, (c) Sensitivity of Pd-ZnO/$ZnCo_2O_4$ HSs towards 5 ppm acetone at 250 °C in dry and humid conditions, (d) Cyclic response of Pd-ZnO/$ZnCo_2O_4$ HSs to 5 ppm of acetone at 250 °C, (e) Dynamic acetone sensing properties to 5 ppm of acetone at 250 °C using 6-day old and 6-month old samples, and (f) Cyclic response to 5 ppm of acetone at 250 °C using 6-month old samples.
